# Dectin-2 Recognizes Mannosylated O-antigens of Human Opportunistic Pathogens and Augments Lipopolysaccharide Activation of Myeloid Cells*

**DOI:** 10.1074/jbc.M116.741256

**Published:** 2016-06-29

**Authors:** Alexandra Wittmann, Dimitra Lamprinaki, Kristian M. Bowles, Ewa Katzenellenbogen, Yuriy A. Knirel, Chris Whitfield, Takashi Nishimura, Naoki Matsumoto, Kazuo Yamamoto, Yoichiro Iwakura, Shinobu Saijo, Norihito Kawasaki

**Affiliations:** From the ‡ Food and Health Institute Strategic Programme, Institute of Food Research, Norwich NR4 7UA, United Kingdom,; § Norwich Medical School, University of East Anglia, Norwich NR4 7TJ, United Kingdom,; the ¶ Ludwik Hirszfeld Institute of Immunology and Experimental Therapy, Wroclaw 53-114, Poland,; the ‖ N. D. Zelinsky Institute of Organic Chemistry, Russian Academy of Sciences, Moscow 119991, Russia,; the ** Department of Molecular and Cellular Biology, University of Guelph, Guelph, Ontario N1G 2W1, Canada,; the ‡‡ Department of Integrated Biosciences, Graduate School of Frontier Sciences, University of Tokyo, Chiba 277-8562, Japan,; the §§ Center for Animal Disease Models, Research Institute for Biomedical Sciences, Tokyo University of Science, Chiba 278-0022, Japan, and; the ¶¶ Department of Molecular Immunology, Medical Mycology Research Center, Chiba University, Chiba 260-8673, Japan

**Keywords:** immunology, lectin, lipopolysaccharide (LPS), polysaccharide, Toll-like receptor 4 (TLR4)

## Abstract

LPS consists of a relatively conserved region of lipid A and core oligosaccharide and a highly variable region of O-antigen polysaccharide. Whereas lipid A is known to bind to the Toll-like receptor 4 (TLR4)-myeloid differentiation factor 2 (MD2) complex, the role of the O-antigen remains unclear. Here we report a novel molecular interaction between dendritic cell-associated C-type lectin-2 (Dectin-2) and mannosylated O-antigen found in a human opportunistic pathogen, *Hafnia alvei* PCM 1223, which has a repeating unit of [-Man-α1,3-Man-α1,2-Man-α1,2-Man-α1,2-Man-α1,3-]. *H. alvei* LPS induced higher levels of TNFα and IL-10 from mouse bone marrow-derived dendritic cells (BM-DCs), when compared with *Salmonella enterica* O66 LPS, which has a repeat of [-Gal-α1,6-Gal-α1,4-[Glc-β1,3]GalNAc-α1,3-GalNAc-β1,3-]. In a cell-based reporter assay, Dectin-2 was shown to recognize *H. alvei* LPS. This binding was inhibited by mannosidase treatment of *H. alvei* LPS and by mutations in the carbohydrate-binding domain of Dectin-2, demonstrating that *H. alvei* LPS is a novel glycan ligand of Dectin-2. The enhanced cytokine production by *H. alvei* LPS was Dectin-2-dependent, because Dectin-2 knock-out BM-DCs failed to do so. This receptor cross-talk between Dectin-2 and TLR4 involved events including spleen tyrosine kinase (Syk) activation and receptor juxtaposition. Furthermore, another mannosylated LPS from *Escherichia coli* O9a also bound to Dectin-2 and augmented TLR4 activation of BM-DCs. Taken together, these data indicate that mannosylated O-antigens from several Gram-negative bacteria augment TLR4 responses through interaction with Dectin-2.

## Introduction

LPS consists of the relatively conserved region of lipid A and core oligosaccharide and the highly variable region of O-antigen polysaccharide (1). The conserved lipid A is recognized by the Toll-like receptor 4 (TLR4)^2^-myeloid differentiation factor 2 (MD2) receptor complex expressed on innate immune cells, such as dendritic cells (DCs) and macrophages (2). Lipid A binding induces TLR4 dimerization and activates further downstream signaling, leading to inflammation-associated expression of genes, such as cytokines and chemokines (3). In addition, previous studies have suggested a regulatory role of the variable O-antigen in LPS activation. For instance, in the LPS-induced sepsis mouse model, disease severity varies, depending on the nature of the O-antigen glycan structure (4). An *in vitro* mechanistic study suggests that the O-antigen affects the kinetics of cytokine production from macrophages (5). Further, a recent report suggests a contribution of O-antigen to the pain occurring during the LPS-induced shock (6).

Glycan-binding proteins (lectins) expressed on the cell surface of innate immune cells have been reported to recognize O-antigens, and the binding may influence TLR4 signaling (7). For example, the macrophage mannose receptor binds to LPS from various *Klebsiella pneumoniae* strains (8); the dendritic cell-specific intercellular adhesion molecule-3-grabbing non-integrin (DC-SIGN) binds to LPS isolated from *Helicobacter pylori* (9); and the Sialic acid binding Ig-like lectin-7 (Siglec-7) binds to lipooligosaccharide of *Campylobacter jejuni* (10).

Dendritic cell-associated C-type lectin-2 (Dectin-2) is a single transmembrane lectin expressed on various myeloid cells in mice and humans, including DCs, monocytes, and macrophages (11–14). Dectin-2 recognizes α-linked mannose structure as a glycan ligand and elicits various cellular responses, including cytokine production (15, 16), cell surface marker induction (17), ligand endocytosis (18), and antigen presentation to CD8T cells (19). The Dectin-2 signaling pathway involves the adaptor molecule Fc receptor common γ-chain (FcRγ) that harbors the immunoreceptor tyrosine-based activation motif (ITAM) in the cytoplasmic domain (16–18). Upon Dectin-2 binding to the glycan ligands, the ITAM motif gets phosphorylated and induces spleen tyrosine kinase (Syk) activation (15, 16). Although glycan ligands of Dectin-2 have been identified in various microbes, including *Candida albicans*, *Malassezia pachydermatis*, and mycobacteria (16, 17, 20, 21), the nature of Dectin-2 ligands from Gram-negative bacteria remains unclear.

According to the microbial polysaccharide database (1), α-linked mannose containing O-antigens are found in various Gram-negative bacterial species, such as *Citrobacter braakii*; *Citrobacter werkmanii* O21 (22, 23); *Escherichia coli* O8, O9, O68, and K12 (24–27); *K. pneumoniae* O3 and O5 (24, 25); *Hafnia alvei* PCM 1223 (28); and *Serratia marcescens* O28 (29). Some of these bacteria can cause nosocomial infections in lung and urinary tract (30–34). Of note, 11% of *K. pneumoniae* clinical isolates were shown to be serotype O3 and O5 (35). Therefore, it is of great importance to determine whether Dectin-2 recognizes the mannosylated O-antigens.

**FIGURE 1.**
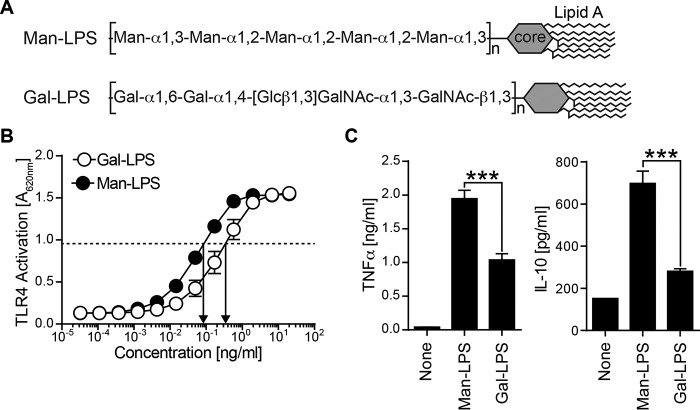
**Comparison of BM-DC response to Man and Gal-LPS.** *A*, two LPS used in this study are shown. Man-LPS from *H. alvei* PCM 1223 has a mannosylated repeating unit, whereas Gal-LPS from *S. enterica* O66 has a galactosylated repeat. *B*, HEK293 cells stably transfected with TLR4-MD2 were cultured in the presence of LPS. The TLR4 activation was monitored by measuring alkaline phosphatase activity using the substrate. *C*, mouse BM-DCs were stimulated with 1 μg/ml Man-LPS or 4 μg/ml Gal-LPS for 7 h. The amount of TNFα and IL-10 in the culture supernatant was analyzed by ELISA. Data are representative of three independent experiments with similar results. *Error bars*, S.D. Statistical analyses were performed by one-way ANOVA followed by Tukey's test. ***, *p* < 0.001.

In this study, we investigated the contribution of the α-linked mannosylated O-antigen in the LPS activation of myeloid cells. We compared DC response and Dectin-2 binding to the mannosylated LPS (Man-LPS) from *H. alvei* PCM 1223 and *E. coli* O9a with the LPS from *Salmonella enterica* O66 or *K. pneumonia* O1, which has the galactosylated O-antigen (Gal-LPS) (Fig. 1*A*). We observed binding between Man-LPS and Dectin-2, which led to augmentation of TLR4 response in mouse DCs and human monocytes. These results demonstrate a novel role of mannosylated O-antigen in activation of TLR4 in myeloid cells.

## Results

### 

#### 

##### Man-LPS Produced a Higher Level of TNFα and IL-10 from Bone Marrow-derived DCs (BM-DCs) than Gal-LPS

To address the contribution of O-antigen in the LPS activation of innate immune cells, we tested two structurally defined LPS. The Man-LPS from *H. alvei* PCM 1223 is built of [-Man-α1,3-Man-α1,2-Man-α1,2-Man-α1,2-Man-α1,3-] repeating units (28), whereas Gal-LPS from *S. enterica* O66 contains [-Gal-α1,6-Gal-α1,4-[Glc-β1,3]-GalNAc-α1,3-GalNAc-β1,3-] repeating units (Fig. 1*A*) (36). The core oligosaccharide and lipid A of these two LPSs are relatively conserved (37–40). TLR4 activation by these two types of LPS was first measured using the TLR4-MD2-expressing HEK293 reporter cells, with Man-LPS being 4-fold more potent as compared with Gal-LPS (Fig. 1*B*). Based on this result, we standardized TLR4 activation by using a 4-fold higher concentration of Gal-LPS compared with Man-LPS in the rest of the study. Under these conditions, Man-LPS induced 2-fold more TNFα and IL-10 from mouse BM-DCs than Gal-LPS (Fig. 1*C*). Man and Gal-LPS induced the co-stimulatory molecule CD80 and mouse MHC class II molecule I-A^b^ to a similar extent (data not shown).

##### Man-LPS Is a Novel Glycan Ligand of Dectin-2

**FIGURE 2.**
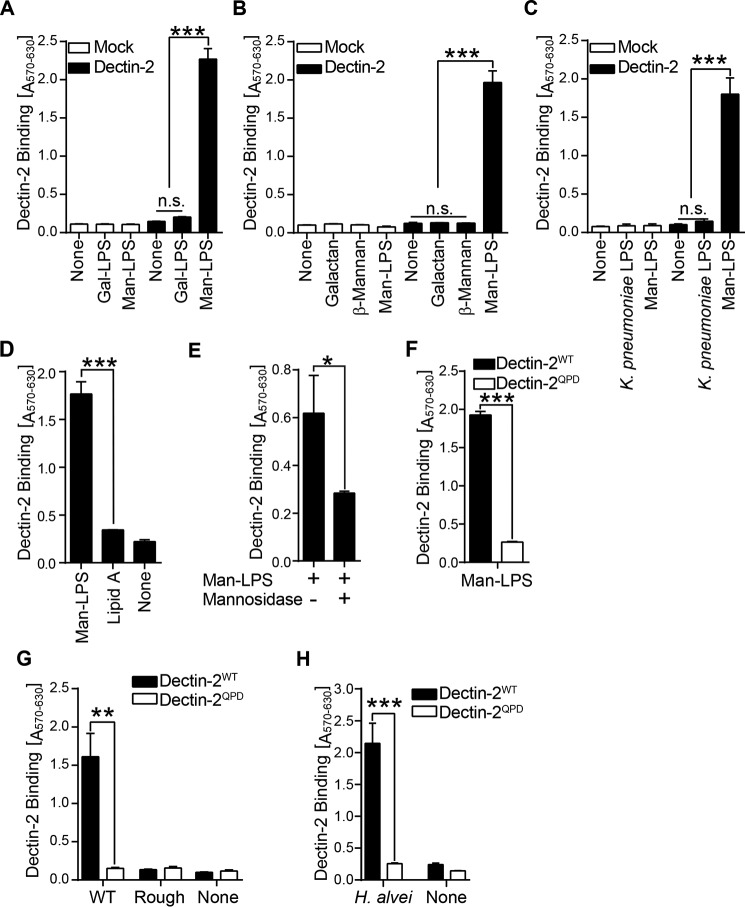
**Dectin-2 recognizes *H. alvei* O-antigen.** *A–D*, mouse Dectin-2 or mock BWZ cells were cultured for 16 h in the 96-well plate coated with purified LPS, plant-derived polysaccharides, lipid A, or nothing. The β-galactosidase activity was measured using the substrate. The data are expressed as the absorbance at 570 nm subtracted with the reference absorbance at 630 nm. *E*, the Man-LPS-coated plate was incubated with the α1-2,3-mannosidase at 37 °C for 14 h. The wells were washed with PBS, and the Dectin-2 reporter cells were added and analyzed as in *A. F–H*, BWZ cells expressing WT Dectin-2 or the QPD mutant were cultured in the presence of Man-LPS, *E. coli* O9a LPS, and the rough mutant LPS or 1.0 × 10^6^ of PFA-fixed *H. alvei* PCM 1223. The binding was monitored as in *A*. Data are representative of three independent experiments with similar results. *Error bars*, S.D. Statistical analyses were performed by one-way ANOVA followed by Tukey's test (*A–D*, *G*, and *H*) or Student's *t* test (*E* and *F*). *, *p* < 0.05; **, *p* < 0.01; ***, *p* < 0.001; *n.s.*, not statistically significant.

Because O-antigen of Man-LPS consists of α-linked mannose, a glycan ligand of Dectin-2, we tested Dectin-2 binding to the purified LPS in a cell-based reporter assay, in which the lectin-glycan interaction is monitored as β-galactosidase expression (41). The Dectin-2 BWZ cells were cultured in a 96-well plate coated with Man and Gal-LPS. In this assay, Dectin-2 bound to Man-LPS but not to Gal-LPS (Fig. 2*A*). No binding was observed between mock BWZ cells and LPS, indicating specific binding of Dectin-2 to Man-LPS (Fig. 2*A*). In addition, plant-derived galactan, β-linked mannan (Fig. 2*B*), and LPS from *K. pneumoniae* O1 (Fig. 2*C*), which has a homopolymeric Gal O-antigen (42), failed to bind to Dectin-2, confirming that Dectin-2 binding to Man-LPS is sugar composition- and linkage-specific rather than nonspecific binding to homopolymeric carbohydrate polymer. Furthermore, the lipid A isolated from Man-LPS failed to bind to Dectin-2 (Fig. 2*D*), and treatment of Man-LPS with α-mannosidase inhibited the binding (Fig. 2*E*), suggesting that binding was mediated by the mannosylated O-antigen of Man-LPS. To assess whether the binding is through the carbohydrate-recognition domain of Dectin-2, we compared the binding of WT Dectin-2 and the QPD mutant that no longer recognizes mannose (17, 43). As shown in Fig. 2*F*, the binding was significantly reduced by the mutations. These results demonstrate that Dectin-2 recognizes the α-linked mannosylated O-antigen of *H. alvei* LPS. Because mannosylated O-antigen is found in other Gram-negative bacteria, including *E. coli* O9a (44), we tested whether Dectin-2 recognizes mannosylated O-antigen from *E. coli* O9a. We found that *E. coli* O9a LPS bound to Dectin-2, whereas the rough mutant LPS, which lacks the O-antigen (45), failed (Fig. 2*G*). We also tested the binding of Dectin-2 to *H. alvei* in the reporter assay. Dectin-2 bound to paraformaldehyde (PFA)-fixed *H. alvei*, whereas the QPD mutant did not (Fig. 2*H*), suggesting the role of Dectin-2 as a recognition receptor for Gram-negative bacteria with α-linked mannosylated O-antigens.

##### Man-LPS Activation Involves a Synergy between Dectin-2 and TLR4

**FIGURE 3.**
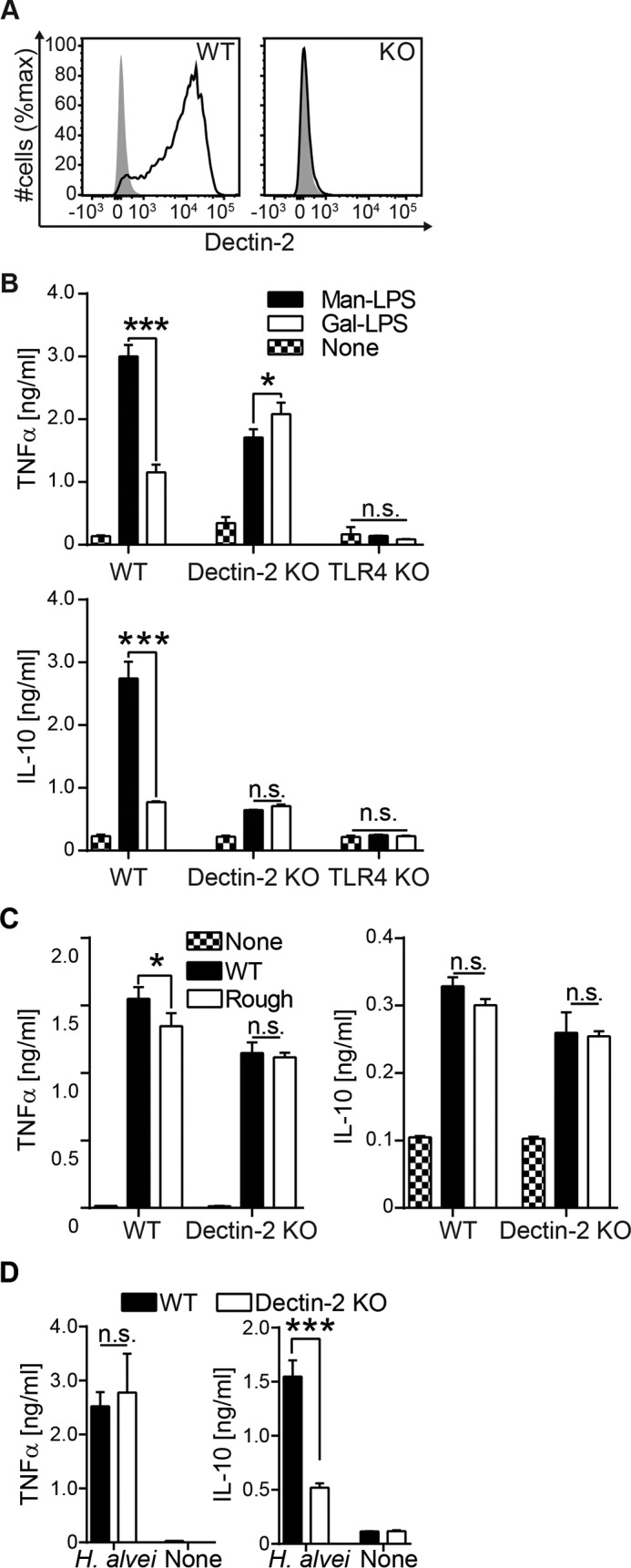
**The binding of Dectin-2 to the O-antigen augments BM-DC response.** *A*, Dectin-2 expression on mouse BM-DCs generated from WT and Dectin-2 KO mice. Cells from *in vitro* culture of bone marrow cells were stained with anti-Dectin-2 (*black*) or isotype (*gray*) control Ab. The stained cells were analyzed by flow cytometry. *B* and *C*, BM-DCs from the indicated background were incubated with the LPS and analyzed for cytokine production as in Fig. 1*C. D*, BM-DCs were incubated with 1.0 × 10^6^ of PFA-fixed *H. alvei* PCM 1223 for 7 h. Cytokine production was monitored by ELISA as in Fig. 1*C*. Data are representative of three independent experiments with similar results. *Error bars*, S.D. Statistical analyses were performed by one-way ANOVA followed by Tukey's test *, *p* < 0.05; ***, *p* < 0.001; *n.s.*, not statistically significant.

To assess the involvement of Dectin-2 in Man-LPS activation of immune cells, we generated BM-DCs from Dectin-2 KO mice (Fig. 3*A*). In contrast to WT BM-DCs, Dectin-2 KO BM-DCs were unable to enhance TNFα and IL-10 production in response to Man-LPS (Fig. 3*B*), indicating that Dectin-2 augments TLR4 activation by Man-LPS. This was reproducible when we used another Gal-LPS from *K. pneumoniae* O1 (data not shown). Of note, in the TLR4 KO BM-DCs, neither Man nor Gal-LPS induced the cytokine production (Fig. 3*B*), suggesting that the mannosylated O-antigen is not sufficient to activate Dectin-2 in the absence of TLR4. Similarly, Dectin-2-dependent enhancement in TNFα production by BM-DCs was seen in response to the WT *E. coli* O9a LPS but not to the rough LPS (Fig. 3*C*). IL-10 response showed a similar tendency, but this was not statistically significant between WT and rough LPS (Fig. 3*C*). The TLR4 reactivity of WT and rough LPS was indistinguishable (data not shown). We also assessed the contribution of Dectin-2 to the DC response toward *H. alvei*. IL-10 production in response to *H. alvei* was Dectin-2-dependent, suggesting a regulatory role of Dectin-2 in the recognition of *H. alvei* (Fig. 3*D*). Although TNFα production in response to Man-LPS was enhanced by Dectin-2 (Fig. 3*B*), the TNFα response to *H. alvei* was similar between WT and Dectin-2 KO BM-DCs (Fig. 3*D*), suggesting alternative molecular mechanisms leading to TNFα production, such as TLR2 that recognizes bacterial cell wall components (46).

##### The Receptor Synergy Is Syk-dependent and Requires Receptor Juxtaposition

**FIGURE 4.**
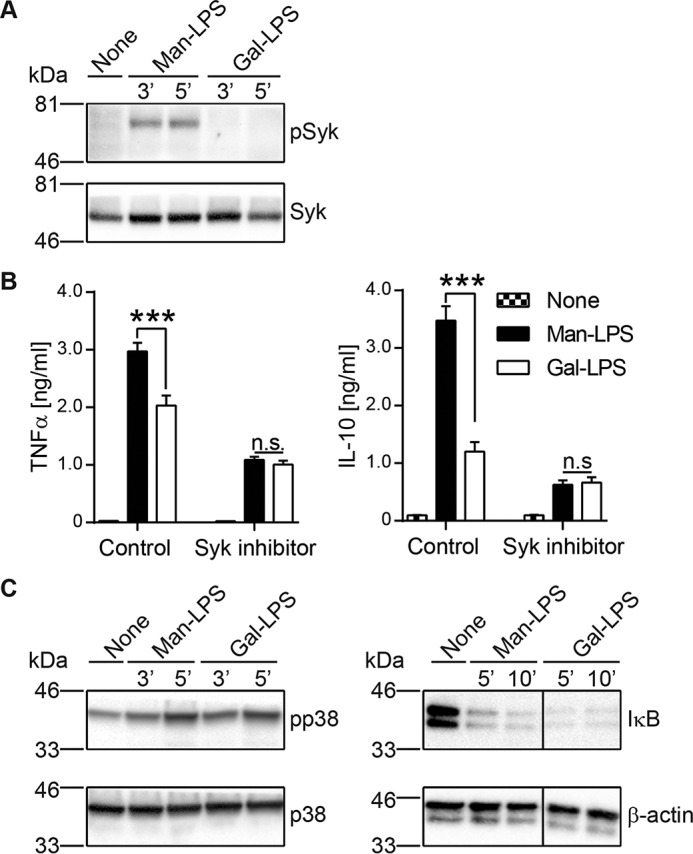
**Syk-dependent BM-DC activation by Man-LPS.** *A*, BM-DCs were stimulated with 1 μg/ml Man-LPS or 4 μg/ml Gal-LPS for the indicated time period. The stimulated cells were lysed and subjected to SDS-PAGE analysis. The proteins were transferred onto nitrocellulose membrane and analyzed for both phosphorylation and expression level of Syk. *B*, BM-DCs were stimulated with LPS in the presence or absence of the Syk inhibitor R406. Cytokine production was monitored by ELISA as in Fig. 1*C. C*, phosphorylation of p38 and degradation of IκB in response to LPS were analyzed as in *A*. Data are representative of three independent experiments with similar results. *Error bars*, S.D. Statistical analyses were performed by one-way ANOVA followed by Tukey's test. ***, *p* < 0.001; *n.s.*, not statistically significant.

To investigate the intracellular signaling events involved in this process, we assessed the impact of Man-LPS on Syk, a key molecule in the Dectin-2 pathway (15, 16). Syk was found to be phosphorylated upon stimulation of BM-DCs by Man-LPS but not Gal-LPS (Fig. 4*A*). Furthermore, treatment of BM-DCs with the Syk inhibitor R406 abrogated the augmented cytokine production in response to Man-LPS (Fig. 4*B*), demonstrating Syk-dependent synergy between Dectin-2 and TLR4. Next, we assessed the impact of the Syk activation by Man-LPS on the activation of NF-κB and MAPK pathways, hallmark of TLR4 activation (3). The phosphorylation of p38 and degradation of IκB was indistinguishable between Man and Gal-LPS, respectively, suggesting that other signaling pathways are modified by Syk activation through Dectin-2 (Fig. 4*C*).

**FIGURE 5.**
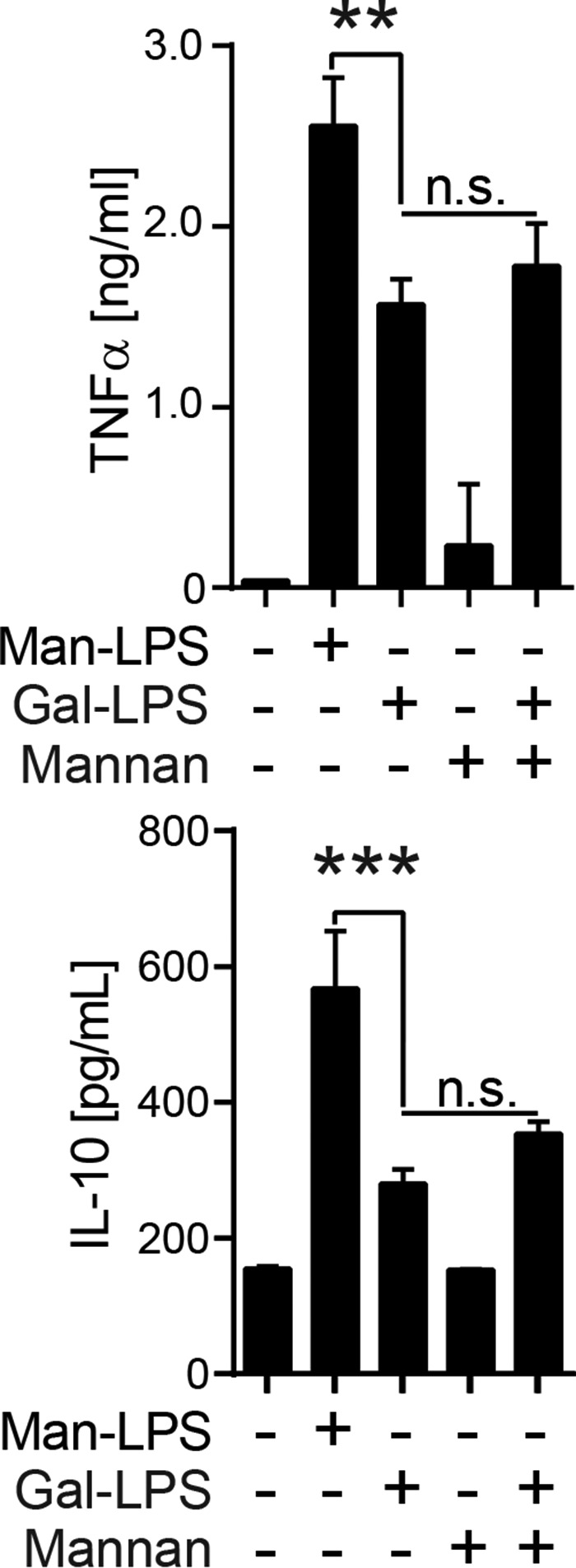
**Co-stimulation of BM-DCs with Gal-LPS and yeast mannan failed to enhance cytokine production.** BM-DCs were stimulated with the indicated stimuli, and cytokine production was analyzed as described in the legend to Fig. 1*C*. Data are representative of three independent experiments with similar results. *Error bars*, S.D. Statistical analyses were performed by one-way ANOVA followed by Tukey's test. **, *p* < 0.01; ***, *p* < 0.001; *n.s.*, not statistically significant.

Because Man-LPS has the binding epitopes for both Dectin-2 and TLR4, we hypothesized that receptor juxtaposition by Man-LPS is the mechanism underpinning the synergy. To test this hypothesis, BM-DCs were stimulated with Gal-LPS as a TLR4 ligand in the presence of yeast α-linked mannan, a known Dectin-2 ligand. As shown in Fig. 5, the addition of yeast mannan was not sufficient to enhance cytokine production, compared with the Gal-LPS, demonstrating that receptor juxtaposition is required to achieve synergy.

##### Human Monocytes Recapitulate the Enhanced Cytokine Production in Response to Man-LPS

**FIGURE 6.**
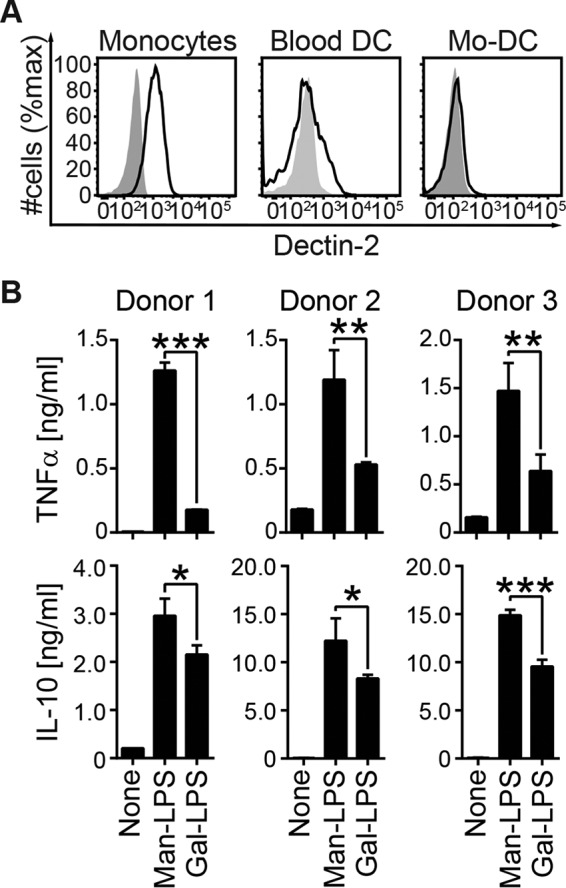
**Human blood monocytes recapitulate the enhanced cytokine response to Man-LPS.** *A*, peripheral blood mononuclear cells or Mo-DCs were stained with anti-Dectin-2 (*black*) or isotype (*gray*) control Ab together with the cell surface markers for each lineage. Human blood monocytes (PI^−^CD14^+^), blood DCs (PI^−^CD3^−^CD14^−^CD16^−^CD19^−^CD20^−^CD56^−^CD11c^+^HLA-DR^+^), and Mo-DCs (PI^−^CD11c^+^) were gated in the analysis. *B*, human blood monocytes were stimulated with the LPS as described in the legend to Fig. 1*C*. Cytokine production was monitored by ELISA. Data are representative of three independent experiments with similar results (*A*) and the results from three individuals (*B*). *Error bars*, S.D. Statistical analyses were performed by one-way ANOVA followed by Tukey's test. *, *p* < 0.05; **, *p* < 0.01; ***, *p* < 0.001.

Man-LPS activation was also tested on human myeloid cells expressing Dectin-2. We found that human peripheral blood monocytes expressed Dectin-2 at a high level, and blood DCs and monocyte-derived DCs (Mo-DCs) expressed at a negligible level (Fig. 6*A*), which is consistent with previous reports analyzing human Dectin-2 mRNA expression among human immune cells (11, 12). Human monocytes produced higher levels of TNFα and IL-10 in response to Man-LPS compared with Gal-LPS (Fig. 6*B*). The anti-Dectin-2 antibody (Ab), however, failed to block the enhanced cytokine production (data not shown), implying the potential involvement of other lectins.

## Discussion

In line with our findings, recent reports show interactions between mammalian lectins and O-antigens. Several strains of *H. alvei* are targeted by Ficolin-3, a complement-associated soluble lectin (47, 48). Langerin, a C-type lectin specific to α-linked mannose, is suggested to recognize the internal Man-α1,2-Man repeat found in O-antigens from *E. coli* O106 and *Shigella boydii* B10 in the pathogen glycan array (49), implying that C-type lectins are capable of interacting with the internal glycan epitopes. At this moment, it is unclear whether the binding of Dectin-2 to the mannosylated O-antigen is mediated by the terminal mannose residue at the non-reducing end, the internal α-linked mannose repeats, or both.

Our findings have identified novel carbohydrate ligands of Dectin-2 and provided a deeper understanding in host-microbe interactions mediated by Dectin-2. Previous studies demonstrate that Dectin-2 plays a key role in fungal and mycobacterial infection and house dust allergy (14, 16, 17, 50). In this report, we have revealed a group of Gram-negative bacteria recognized by Dectin-2. LPS from *H. alvei* PCM 1223 and *E. coli* O9a bound to Dectin-2 and enhanced TLR4 responses of BM-DCs in a Dectin-2-dependent manner, suggesting a novel role of Dectin-2 in the interaction between host and Gram-negative bacteria bearing α-linked mannosylated O-antigen. Of note, *H. alvei* LPS was more potent than *E. coli* O9a LPS in Dectin-2 engagement in BM-DCs (Fig. 3, *B* and *C*); this may be due to the different O-chain length because the sugar structure is identical between these two bacteria (44).

Our findings that Dectin-2 interacts with TLR4 upon Man-LPS stimulation strengthen the proposed model that lectins are capable of regulating TLR pathways in various ways. For instance, stimulation of DCs and macrophages with β-glucan in the presence of various TLR ligands allowed enhancement of TNFα and IL-10 production (51–53). Co-stimulation of DCs with DC-SIGN and TLR ligands selectively enhances IL-10 production (54, 55). Although these studies clearly demonstrate the cross-talk between C-type lectins and TLRs, the molecular mechanisms underpinning such receptor cross-talk remain elusive. One potential mechanism is receptor juxtaposition, as previously proposed for the ITAM-coupled lectin Siglec-H; positioning Siglec-H in close proximity to TLR9 in the endosome would enhance the TLR9 activation (56). Here we showed that such synergy was observed between Dectin-2 and TLR4. Whether this is a common strategy for the modulation of TLR functions by membrane-bound lectins remains to be demonstrated.

We have identified Syk as a key molecule for cross-talk between Dectin-2 and TLR4. Syk has been proposed as a regulator of TLR4 signaling. Several studies have reported that in DCs and macrophages, Syk gets phosphorylated upon LPS stimulation (57), and Syk deficiency results in enhanced TNFα and reduced IL-10 (58). Of note, most of the studies employed LPS from *E. coli* O111:B4 (59). In our study, because Gal-LPS failed to induce phosphorylation of Syk, it is likely that O-antigen structure influences Syk activation. Thus, to address the role of Syk in TLR4 signaling, it would be essential to test whether reported Syk activation by *E. coli* O111 LPS involves Dectin-2 or other lectins.

**FIGURE 7.**
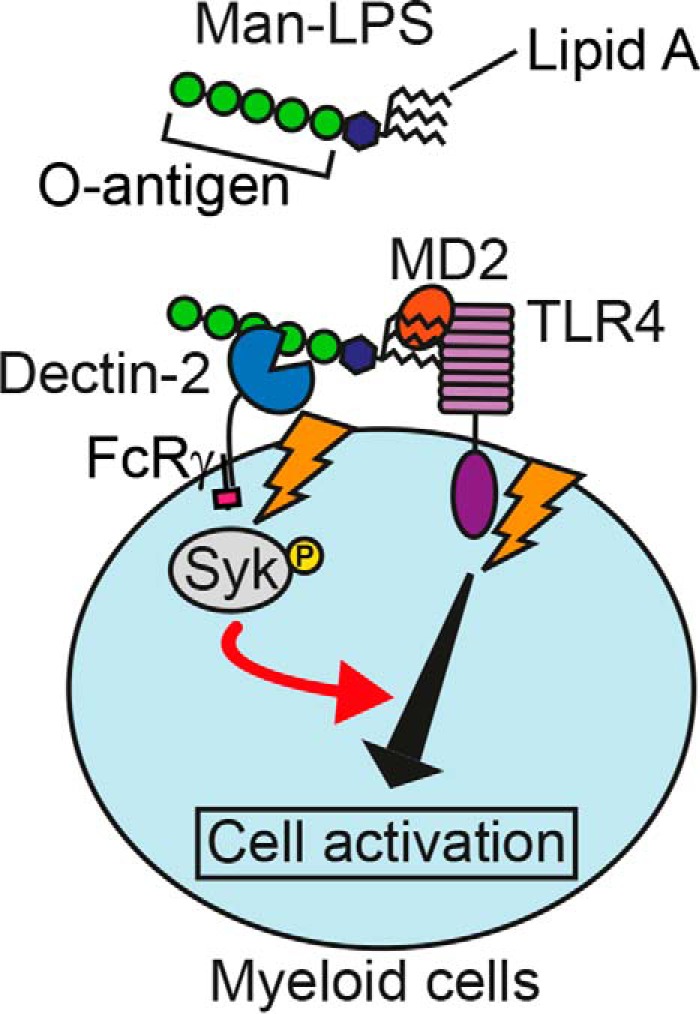
**Role of mannosylated O-antigen in LPS activation of immune cells.** Man-LPS is recognized by both Dectin-2 and TLR4, leading to activation of Syk. The Syk activation results in enhanced TLR4 responses, such as cytokine production. The signaling pathways affected by Syk are yet to be determined.

Although our findings have revealed a novel function of mannosylated O-antigen in LPS activation of innate immune cells (Fig. 7), the role of core oligosaccharides in the TLR4 activation remains elusive. In this regard, SIGNR1 is reported to recognize the core oligosaccharide of *E. coli* LPS and augment cytokine responses (60). Further studies of lectin recognition of both highly variable O-antigen and the conserved core oligosaccharide are warranted to gain a better understanding of LPS recognition by innate immune cells.

## Experimental Procedures

### 

#### 

##### Mice

C57BL/6J (WT), TLR4 KO (a gift from Dr. J. S. Frick, University of Tuebingen), and Dectin-2 KO mice were maintained in the specific pathogen-free animal facilities at the University of East Anglia, University of Tuebingen, and Chiba University, respectively. Animal use in this study was in accordance with United Kingdom Home Office guidelines, the Regierungspraesidium Tuebingen, and the ethics committee of Chiba University.

##### Reagents

All of the chemical reagents were obtained from Sigma-Aldrich, unless otherwise stated. Abs used in the flow cytometry were obtained from Biolegend, unless otherwise indicated, and include those against mouse CD80 (GL-1), I-A^b^ (AF6–120.1), human Dectin-2 (R&D systems, 545943), CD3 (OKT3), CD14 (M5E2), CD16 (3G8), CD19 (HIB19), CD20 (2H7), CD56 (HCD56), CD11c (3.9), and HLA-DR (L243). The Abs used for Western blot were obtained from Cell Signaling and include Syk (D3Z1E), phospho-Syk (C87C1), p38 (D13E1), phospho-p38 (D3F9), IkB (rabbit polyclonal Ab), and β-actin (13E5). LPS from *H. alvei* PCM 1223, *S. enterica* O66, *K. pneumoniae* O1, *E. coli* O9a, and the rough mutant were isolated as described previously (28, 42, 45, 61, 62). The lipid A from the Man-LPS was isolated by hydrolysis as described previously (28). Galactan and β-linked mannans were from Megazyme. The Syk inhibitor R406 was purchased from InvivoGen. ELISA kits for mouse and human TNFα and IL-10 were from Biolegend and were used according to the manufacturer's instructions.

##### Cells and Bacteria

BWZ.36 cells harboring IL-2-driven β-galactosidase cassette (63) and the retrovirus-packaging cell line Plat-E were obtained from Dr. N. Shastri (University of California, Berkeley, CA) and Dr. T. Kitamura (University of Tokyo), respectively, and maintained as described before (64). *H. alvei* PCM 1223 was obtained from the Polish Collection of Microorganisms and cultured in Luria broth at 37 °C under shaking at 200 rpm (Innova 44 incubator, New Brunswick Scientific).

##### Flow Cytometry

Cells were washed with Hanks' balanced saline solution (Lonza) containing 0.1% BSA, 2 mm EDTA (FACS buffer); blocked with anti-mouse Fc block Ab (Biolegend) for 5 min at 25 °C; and stained with the Abs for 30 min at 4 °C. Stained cells were washed once with FACS buffer and analyzed by Fortessa (BD Biosciences). For dead cell exclusion, propidium iodide (PI) was added to the sample at a final concentration of 0.33 μg/ml before the analysis. Acquired data were analyzed with FlowJo (Tree Star).

##### TLR4 Reporter Assay

The TLR4 reporter assay was performed using the HEK-Blue human TLR4 reporter cells that produce alkaline phosphatase in response to LPS, according to the manufacturer's instructions (InvivoGen). Briefly, the TLR4-HEK293 cells were cultured in the HEK-Blue detection medium containing the substrate for alkaline phosphatase in the presence of the LPS for 20 h. After incubation, the absorbance at 620 nm was measured.

##### Establishment of Mouse Dectin-2 Reporter Cells

The Dectin-2 reporter cells were established as described previously (64). Briefly, the extracellular domain of mouse Dectin-2 (Gln^42^–Leu^209^) was cloned into the retrovirus vector pMXs-IRES-EGFP-Ly49A-CD3ζ harboring the transmembrane region of the mouse Ly49A and the cytoplasmic domain of the mouse CD3ζ (64). The pMXs-IRES-EGFP-Dectin-2-Ly49A-CD3ζ vector and the parental vector were used to establish Dectin-2 and mock BWZ cells, respectively, for the retrovirus transduction using Plat-E cells (65). To establish reporter cells expressing the carbohydrate-binding incompetent mutant of Dectin-2 (Dectin-2^QPD^), two missense mutations (G502C and A508G) in the mannose recognition domain were introduced, which results in amino acid substitutions E168Q and N170D. The DNA fragment encoding the extracellular domain of Dectin-2 with the two missense mutations was synthesized (GenScript) and cloned into the pMXs-IRES-EGFP-Ly49A-CD3ζ vector and used as described above.

##### Dectin-2 Reporter Assay

A reporter assay was performed as described previously (64). Briefly, the 96-well flat-bottom ELISA plate (MaxiSorp, Thermo Scientific Nunc) was coated with 100 μl containing 40 ng of LPS or polysaccharides in 100 mm sodium bicarbonate buffer, pH 9.5, for 16 h at 4 °C. Dectin-2 or mock BWZ cells were then cultured in the well, and β-galactosidase activity was monitored as described previously (64). For the mannosidase treatment, a 96-well plate was coated with 1.6 ng of Man-LPS. After discarding the solution, wells were washed once with 200 μl of PBS and blocked with 100 μl of 4% BSA in PBS for 1 h at 25 °C. Blocked wells were washed, and 50 units of α1–2,3 mannosidase (New England BioLabs) was added to the wells and incubated at 37 °C for 14 h. The reporter cells were added to test the binding. For the reporter assay with bacteria, 1.0 × 10^6^ of PFA-fixed *H. alvei* were immobilized on the 96-well plate as described above.

##### Generation of Anti-Dectin-2 mAb

The anti-Dectin-2 mAb was generated as described previously (66). Briefly, two female Lewis rats (Japan SLC) were immunized with Dectin-2-BWZ cells emulsified with complete Freund's adjuvant (Difco). Following two injections of the cells emulsified with incomplete Freund's adjuvant (Difco Laboratories), the immunized rats were sacrificed, and common iliac lymph nodes were harvested to generate hybridomas as described previously (66). The established hybridomas were screened by the reporter assay described previously (67). The animal experiments were performed in accordance with the institutional animal ethics committee at the University of Tokyo. The established hybridoma clone 2B4 produced anti-mouse Dectin-2 mAb, a rat IgG2a, κ chain determined by flow cytometry (66). Monoclonal Ab 2B4 was purified from the culture supernatant and labeled with Alexa Fluor-647 (Life Technologies, Inc.) as described previously (66).

##### Generation of BM-DCs

BM-DCs were generated by *in vitro* culture of mouse bone marrow cells as described previously (68). Briefly, 3 × 10^6^ mouse bone marrow cells were cultured in a 10-cm dish with 12 ml of the RPMI 1640 medium (Lonza) supplemented with 25 mm HEPES, 10% FBS (Thermo Scientific Gibco), 55 μm 2-mercaptoethanol, 100 units/ml penicillin and 100 μg/ml streptomycin (Lonza), 2 mm glutamine (Lonza), 1 mm non-essential amino acids (Lonza), 1 mm sodium pyruvate (Lonza), and 20 ng/ml mouse GM-CSF (Peprotech). The culture was kept undisturbed for 6 days. On day 6, the cells were harvested and used as BM-DCs.

##### Stimulation of BM-DCs

BM-DCs (1 × 10^5^ cells) were cultured in a 96-well plate using the RPMI medium described above without GM-CSF. Man and Gal-LPS were added to the culture at a final concentration of 1 or 4 μg/ml, respectively, and incubated for 7 h at 37 °C. The concentration was standardized for their reactivity toward TLR4 (Fig. 1*B*). For the cytokine analysis, culture supernatant was harvested, and TNFα and IL-10 were measured by ELISA. When the Syk inhibitor R406 was used, it was added to the cell culture at a final concentration of 1 μm and incubated for 30 min before the addition of LPS. For CD80 and I-A^b^ expression analysis, stimulated BM-DCs were harvested and analyzed by flow cytometry as described above.

##### Western Blotting of Intracellular Proteins

One million BM-DCs were stimulated with 1 or 4 μg/ml Man- and Gal-LPS, respectively, for the indicated time period. Cells were then processed as described previously with adaptation of lysis buffer volume to 75 μl (69). Cell lysate equivalent to two hundred thousand cells (15 μl) was subjected to SDS-PAGE using a 4–15% gradient TGX minigel (Bio-Rad) for 90 min at 100 V. Proteins were transferred onto nitrocellulose membrane (GE Healthcare) at 100 V for 30 min. The membranes were blocked with 5% nonfat milk (Lonza) in PBS containing 0.05% Tween 20 (PBS-T) for 1 h at 25 °C. The blocked membranes were washed four times by incubating in PBS-T for 5 min each. Membranes were then incubated with the primary antibodies in PBS containing 1% bovine serum albumin for 1 h at 25 °C at a dilution of 1:1000 for Syk, phospho-Syk, IκB, and β-actin and 1:5000 for p38 and phospho-p38. Membranes were washed as above and probed with anti-rabbit IgG conjugated with horseradish peroxidase (Cell Signaling) in 5% nonfat milk in PBS-T at a dilution of 1:3000 for 1 h at 25 °C. Membranes were washed as above and incubated with the ECL detection reagent (GE Healthcare) and imaged using Fluorochem E (ProteinSimple).

##### Human Blood Monocytes

Human peripheral blood was obtained from the hemochromatosis patients undergoing a therapeutic venesection at the Norfolk and Norwich University Hospital (Norwich, UK). Human blood monocytes were isolated as described previously (70). The Dectin-2 expression on the monocytes was analyzed by flow cytometry. The freshly isolated monocytes were stimulated with 1 ng/ml Man and 4 ng/ml Gal-LPS for 20 h at 37 °C. Human TNFα and IL-10 production was monitored by ELISA. Mo-DCs were generated by *in vitro* culture of human blood monocytes as described previously (70). Blood collection in this study was approved by the ethics committee at the Faculty of Medicine and Health Sciences at the University of East Anglia (reference number 2013/2014-14HT). Importantly, the monocytes in the patients with iron overload have been shown to respond to LPS, although the response was lower when compared with that of monocytes from healthy donors (71), justifying the use of monocytes from the patients to study human Dectin-2 function.

##### Statistical Analysis

Student's *t* test and one-way ANOVA followed by Tukey's test were used for statistical analysis on Prism software (GraphPad). *p* < 0.05 was considered as statistically significant.

## Author Contributions

A. W. and N. K. conceived and coordinated the study and wrote the paper. A. W. designed, performed, and analyzed all of the experiments. D. L. performed and analyzed the experiments in Figs. 3–5. K. M. B. arranged human blood sample collection. E. K. and Y. A. K. provided *H. alvei* and *S. enterica* LPS, respectively. C. W. provided LPS from *E. coli* O9a, the rough mutant, and *K. pneumoniae* O1. Y. I. and S. S. provided Dectin-2 KO bone marrow cells. T. N. and N. K. established the Dectin-2-BWZ cells and generated the anti-Dectin-2 mAb. N. M. and K. Y. conceived and coordinated establishment of Dectin-2-BWZ cells and generation of the anti-Dectin-2 mAb. All authors analyzed the results and approved the final version of the manuscript.

## Footnotes

2 The abbreviations used are:

TLR: Toll-like receptor
Ab: antibody
DC: dendritic cell
BM-DC: bone marrow-derived dendritic cell
DC-SIGN: dendritic cell-specific intercellular adhesion molecule-3-grabbing non-integrin
Dectin-2: dendritic cell-associated lectin-2
FcRγ: Fc receptor common γ-chain
Gal: galactose
Gal-LPS: galactosylated LPS
GalNAc: *N*-acetylgalactosamine
Glc: glucose
Man: mannose
Man-LPS: mannosylated-LPS
MD2: myeloid differentiation factor 2
Mo-DCs: monocyte-derived DCs
PFA: paraformaldehyde
PI: propidium iodide
Siglec: sialic acid binding Ig-like lectin
Syk: spleen tyrosine kinase
ANOVA: analysis of variance.
